# Application of DTI and fMRI in moyamoya disease

**DOI:** 10.3389/fneur.2022.948830

**Published:** 2022-08-05

**Authors:** Xiaokuan Hao, Ziqi Liu, Shihao He, Yanru Wang, Yuanli Zhao, Rong Wang

**Affiliations:** ^1^Department of Neurosurgery, Beijing Tiantan Hospital, Capital Medical University, Beijing, China; ^2^China National Clinical Research Center for Neurological Diseases, Beijing, China; ^3^Center of Stroke, Beijing Institute for Brain Disorders, Beijing, China

**Keywords:** moyamoya disease (MMD), diffusion tensor imaging (DTI), functional MRI (fMRI), brain network, white matter fiber bundles

## Abstract

Moyamoya disease (MMD) is a chronic and progressive cerebrovascular stenosis or occlusive disease that occurs near Willis blood vessels. Diffusion tensor imaging (DTI) and functional magnetic resonance imaging (fMRI) are used to detect the microstructure of white matter and the function of gray matter, respectively. The damage of these structures will lead to the change of cognitive level in patients with moyamoya disease. In this paper, the principles of DTI and fMRI, their applications and challenges in moyamoya disease are reviewed.

## Introduction

Moyamoya disease (MMD) is an uncommon cerebrovascular disease which leads to progressive stenosis and occlusion of the bilateral internal carotid artery and main intracerebral arteries, with subsequent abnormally formed collateral vessels 90 (1). It was first described by Takeuchi and Shimizu in 1957 and then termed by Suzuki and Takaku's in 1969 (2). Diffusion tensor imaging (DTI) and functional magnetic resonance imaging (fMRI) techniques have made remarkable achievements in cognitive and cerebrovascular disease (3, 4), and can be used to examine white matter (WM) microstructure and gray matter (GM) function in patients with MMD, respectively. In addition, there has been evidence that the cognitive level of patients with MMD is related to white matter damage (5), and the quantitative value of patients' cognitive level reflected in fMRI will also change after revasculopathy surgery (6). Therefore, it is of fundamental and clinical significance to discuss the application of these two neuroimaging techniques in the study of brain damage in patients with MMD.

## Fundamentals and background

### The principle and parameters of DTI

**Figure 1:**
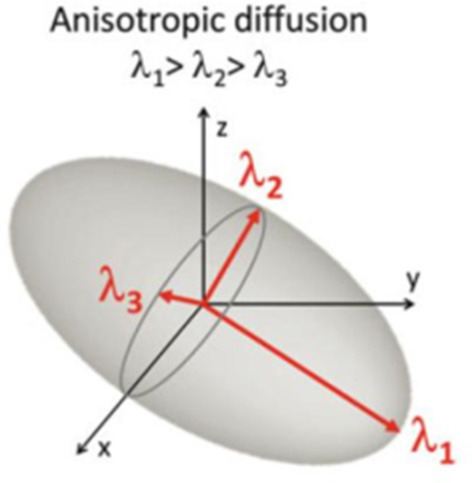
Diffusion coefficients.

Diffusion-weighted imaging (DWI) is a quantitative technique that utilizes the diffusion of water in biological tissues (7). The diffusion index is used to measure the difficulty of water molecules horizontal movement. In biological tissue, since various structures of the tissue (cell membrane, myelin sheath, etc.) impede the free movement of water molecules (7), the diffusion coefficient is much lower than that in free water. Diffusion tensor imaging (DTI) is a non-invasive imaging method developed on the basis of DWI for the study of white matter fiber bundle injury (8). The diffusion distance of water molecules in each gradient direction is measured by increasing the diffusion sensitivity coefficient (B value) and increasing the numbers of gradient directions, so that the eigenvalue can be calculated by using the difference of the diffusion tensor in different tissues. In general, water molecules move or diffuse much faster parallel to the white matter fibers than they do perpendicular to them. Therefore, in each voxel of the fiber bundle, if the diffusion tensor is regarded as an ellipsoid (9), the maximum diffusion coefficients parallel to the direction of the fiber are defined as λ_1_, and those perpendicular to the direction of the fiber are defined as λ_2_ and λ_3_ (λ_1_ > λ_2_ > λ_3_) (Figure 1). Through λ_1_, λ_2_, λ_3_, we can calculate the coefficients of different DTI scans, such as fractional anisotropy (FA), mean diffusivity (MD), radial diffusivity (RD), axial diffusivity (AD) values.

FA refers to the partial anisotropy coefficient, which is the proportion of anisotropic components of water molecules in the whole diffusion, and its value ranges from 0 to 1. The closer FA value is to 0, the more unrestricted the movement of water molecules are, and the closer it is to free water, the higher the anisotropy is. The FA value in CSF is close to 0, while tissues such as white matter fiber bundles, which strictly constrain the direction of movement of water molecules, the FA value is close to 1. A high degree of myelination in white matter causes axons to gather more closely together, increasing the value of FA. On the contrary, axon damage, demyelination, increased membrane permeability, and decreased density and number will decrease FA value (10–12). The values of AD and RD also have similar significance.


$$\begin{array}{l} FA=\frac{\sqrt{3\left({\left(\lambda_{1}-\lambda \right)}^{2}+{\left(\lambda_{2}-\lambda \right)}^{2}+{\left(\lambda_{3}-\lambda \right)}^{2}\right)}}{2\left(\lambda_{1}^{2}+\lambda_{2}^{2}+\lambda_{3}^{2}\right)} \end{array}$$

In order to comprehensively evaluate the diffusion of a certain element or region, the influence of anisotropic diffusion must be eliminated and represented by a parameter (MD) whose change does not depend on the direction of diffusion. MD reflects the diffusion level of the whole molecule (i.e., the size of the mean ellipsoid) and the diffusion resistance of the whole molecule. It indicates only the magnitude of the diffusion, not the direction. In general, the MD value is lower in white matter, but higher in ventricles where the movement of water molecules is not limited (13). The larger the MD, the more free water molecules there are in the tissue.


$$\begin{array}{l} MD=\frac{\lambda_{1}+\lambda_{2}+\lambda_{3}}{3} \end{array}$$

### Basic principles and background of FMRI

Blood oxygen level dependent-functional magnetic resonance imaging (bold-fMRI) is an imaging technology developed since the 1990s designed to study brain function (14). Its imaging theory mainly uses the change of local magnetic field property caused by the mismatch between the increase degree of local cerebral blood flow and oxygen consumption. Both regional cerebral blood flow and oxygen consumption increase when neurons are generating electrical activity, but the increase of cerebral blood flow was more than that of oxygen consumption. This difference results in a relative reduction in the concentration of paramagnetic deoxygenated hemoglobin (15). Deoxyhemoglobin has the effect of shortening T2 signal, and its reduction will lead to the decrease of shortening T2. Compared with the resting state, T2 in local brain regions is relatively prolonged, so it shows an enhanced signal on T2 weighted functional magnetic resonance imaging (16). Therefore, bold-fMRI can be used to indirectly observe the activity of neurons and even the connectivity of functional areas of the brain.

The blood oxygen level dependence (BOLD signal) was first proposed by Ogawa et al. in 1990 (17). He proposed that the change of blood oxygen level in the brain will lead to the change of local magnetic field uniformity, resulting in the obvious change of NMR signal, which is called BOLD signal. In 1991, research teams from Minnesota and Massachusetts General Hospitals obtained the first successful fMRI results using BOLD comparisons (13). The results were presented orally at the Magnetic resonance conference in San Francisco in August 1991. Subsequently, in 1992 and 1993, experimental results based on BOLD brain functional imaging were obtained in various laboratories (13). Since then, the research boom of functional magnetic resonance imaging has started. In the past few decades, especially tasking-state functional magnetic resonance imaging (ts-fMRI) has been widely used as the benchmark method to locate and map the brain functional specialized areas under the stimulation of specific tasks (18).

At present, there are two main parameters that reflect the attributes of BOLD signal area: one is low frequency fluctuation amplitude (ALFF), which measures the signal intensity in low frequency oscillation in spontaneous nerve activity (19). ALFF is correlated with the potential activity of local brain regions (20), and the amplitude of oscillation can be used as an indicator to detect changes in neural function (21). The second is regional homogeneity (ReHo), which reflects the statistical similarity of local neural activity between adjacent regions of space (22). Both methods have been widely used in the assessment of local neurological function in neurological and neuropsychiatric disorders (23, 24).

In recent years, resting state functional magnetic resonance imaging (resting-state fMRI, rs-fMRI) has been widely used to study the functional connections between different regions of the brain. In the resting state, the spontaneous BOLD signal fluctuations between the relevant brain regions have spatial synchronization, which has been used to find a variety of resting state functional connection networks (25).

## Application of DTI and FMRI in moyamoya disease

### Development and use of DTI in MMD

**Figure 2:**
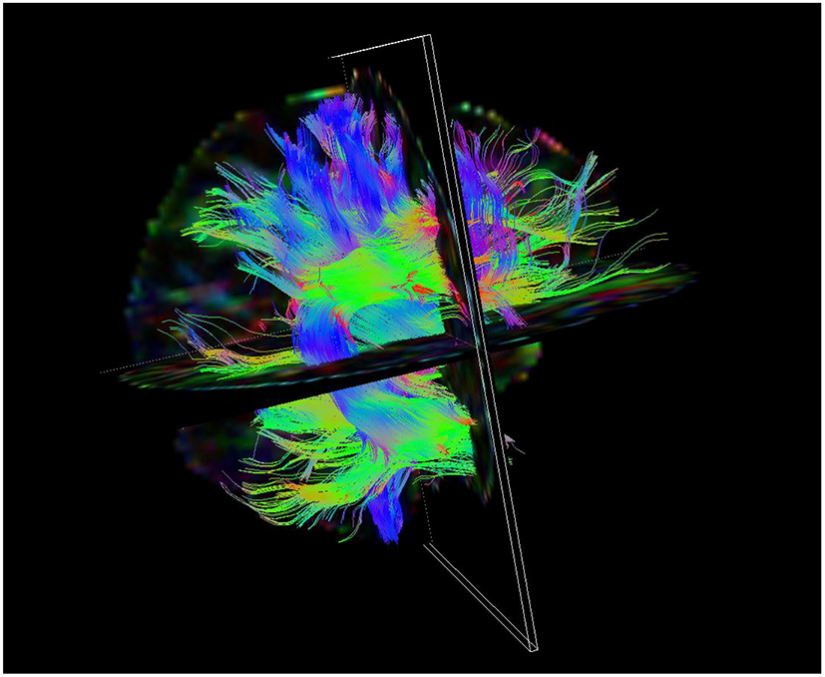
Picture of white matter fiber bundle tracking results in patients with MMD by DTI.

Figure 2 is the white matter fiber bundle tracking result of patients with MMD by DTI. The understanding of white matter fiber bundle damage in MMD by DTI technique is gradual. Initially scientists focused on the differences between the infarcted and normal brain or between the infarcted and non-infarcted hemispheres. This difference was confirmed in a study of cerebral infarction patients with MMD. Nobuyuki Mori et al. (26) found that there was a significant difference in whole-brain histogram (WBH) diffusion tensor imaging between MMD and normal volunteers, while there was no significant difference in WBH-diffusion tensor imaging between MMD patients without infarction and normal control group. The authors suggest that no significant damage to brain tissue occurs in ischemic MMD without infarction. However, Statistical Parametric Mapping (SPM2), the voxel-based analysis software used at that time, was relatively unadvanced in image processing, and the author did not study specific brain regions in the analysis of the whole brain, which had certain limitations.

With the deepening of research, many researchers have found that even in patients with ischemic MMD without infarction, there is still latent white matter damage, which is similar to DTI can detect the degree of nerve and white matter damage in patients with consciousness disorders with high sensitivity (27). In 2011, Jeong et al. (28) used the regions of interest (ROI) to compare the FA value and Apparent diffusion coefficient (ADC) value of 20 patients with normal white matter MMD and 20 age-matched control group's centrum semiovale. The results showed that the FA and ADC value of patients were significantly reduced. Moreover, FA and ADC values were lower in the cerebral hemispheres with delayed peak time in MMD (the hemisphere with more severe ischemia). The authors hypothetically propose from a DTI perspective that the normal presentation white matter of MMD without cerebral infarction may be affected by chronic hypoperfusion, resulting in cumulative microstructural damage that is not visible on conventional MRI. However, the ROI of the centrum semiovale extracted by the author is too rough to be unified. Therefore, in the later studies, researchers have found new methods to solve this problem.

In 2014, Kazumata et al. (29) used Tract-Based Spatial Statistics (TBSS) to analyze white matter of 23 asymptomatic MMD patients and 23 controls, and combined it with cognition function. They found that the FA of white matter tracts in the lateral prefrontal area, cingulate area and inferior parietal area was significantly correlated with processing speed, executive function (attention) and working memory. This study combined cognitive impairment with damage to white matter fibers in asymptomatic MMD patients and found a correlation between cognitive impairment and white matter damage. Similarly, in the subsequent study of Liu et al. (5), cognitive test and TBSS analysis were also conducted on 14 asymptomatic patients with MMD, and it was found that left brain uncinate fasciculus (UF) and inferior fronto-occipital fasciculus (IFO) may be the key brain regions affecting computing function, while bilateral brain IFO regions may affect intelligence. RD and AD may be better early predictors of chronic white matter injury than FA, while MD tends to have overall indirect changes.

After the study of pre-operation white matter damage in MMD became more and more clear, Kazumata et al. (30) studied the recovery of white matter and perfusion in 17 patients with MMD in a short period (within 14 days) after bilateral superficial temporal artery-middle cerebral artery (STA-MCA) revascularization in 2017. The authors found that regional cerebral blood flow (rCBF) in the lateral prefrontal cortex increased gradually during the first week postoperatively. FA and AD decreased in the anterior and posterior limbs of the internal capsule during the first and second days and the third and sixth days. RD increased most significantly. FA, RD and AD returned to their preoperative levels on day 14. On the one hand, it confirmed the importance of perfusion for white matter injury in MMD patients, and post-operative hyper-perfusion may damage white matter temporarily. On the other hand, the STA-MCA revascularization in MMD patients do not show short-termed benefits for white matter recovery. However, the effect of the revascularization on long-term white matter recovery remains unclear. Therefore, Kazumata et al. (31) further studied the cognitive recovery and white matter fiber recovery in 25 patients with MMD who underwent bilateral STA-MCA revascularization in 2019. The results showed that there were significant changes in performance intelligence quotient (PIQ) and perceptual organization (PO) after operation. The FA value of the anterior bundle of bilateral superior longitudinal tract (SLF) gradually increased after surgery and reached statistically significant after 2–4 years, and was positively correlated with the recovery rate of PIQ and PO. Here, preoperative injury and postoperative repair of the white matter fibrous tracts in MMD were linked, and an association between white matter injury in MMD and cerebral perfusion was found, as well as between white matter injury and cognitive impairment.

It is generally believed that the formation of myelin sheath limits the developmental changes and plasticity of axons (32). Thus, delayed myelination may be one of the reasons why the brain's advanced abilities continue to grow even during adulthood (10). As the brain matures, structures such as cells and axon membranes become denser, and the mobility of water molecules was increasingly limited. With the development of white matter, the changes of water diffusion perpendicular to white matter fiber may stand for the change of the myelin sheath width (33), and indirectly caused the RD of DTI parameters change. Therefore, for the affected by long-term hypoperfusion, white matter injury in MMD, especially the damage to the myelin sheath or dysplasia, is more likely to be one of significant reasons for cognitive dysfunction; The time-dependent improvement in perfusion after revascularization may be the possible reason for the improvement in cognitive function through restoration of white matter function, whereas short-term post-operative hyper-perfusion may injury white matter function, temporarily. This is a major achievement of DTI research on MMD in recent years. More article details could be found in Table 1.

**Table 1:** Studies in recent years about DTI analysis of MMD.

	**Time**	**Author**	**Key point**	**Observe indicators**	**DTI analysis method**	**Other design methods**	**Patient selection**
Pre-operation	2008	Nobuyuki Mori	MMD with infarction exist white matter injuries while MMD without infarction do not.	peak height FA, peak height MD	SPM2	None	15 with and 12 without infarction
							
	2011	H. Jeong	Chronic hemispheric hypoperfusion results in damage to white matter (centrum semiovale)	FAcs, ADCcs	NeuRoi	rCBF, DWI	20 asymmetric
	2014	Ken Kazumata	FA in the LP, Cingulate and IPL was correlated with processing speed, executive function and working memory	FA, MD, AD, RD	FSL	FSL-VBM, Cognition test	23 asymmetric
	2016	Ken Kazumata	DKI can also show the microstructural changes of deep white matter prone to ischemia and solve the problem of fiber crossover	FA, MD, AD, RD	FSL	DKI	23 without infarction
	2020	Ziqi Liu	Left UF and IFO may affect arithmetic function. Bilateral IFO effect intelligence. RD and AD may be better indicators for early prediction.	FA, MD, AD, RD	FSL-TBSS	Cognition test	14 asymmetric
Post-operation	2017	Ken Kazumata	rCBF and FA, AD, RD gradually return to pre-operation level in 2 weeks. Revascularization surgery may temporarily damage subcortical structures due to hyperperfusion	FA, MD, AD, RD	FSL, SPM8	DKI, rCBF, SPM8	17 patients
	2019	Ken Kazumata	Gradual increases in FA in the bilateral SLF at 2–4 years after surgery. Revascularization surgery may improve processing speed and attention.	FA, MD, AD, RD	FSL-TBSS	SPM12, Cognition test	25 asymmetric

LP, lateral prefrontal; IPL, inferior parietal lobes; UF, uncinate fasciculus; ILF, inferior longitudinal fasciculus; SLF, superior longitudinal fasciculus; FA, Fractional anisotropy; MD, Mean diffusivity; RD, Radial diffusivity; AD, Axial diffusivity; cs, centrum semiovale; ADC, apparent diffusion coefficient; rCBF, regional cerebral blood flow; TBSS, Tract-Based Spatial Statistics; DWI, Diffusion-weighted imaging; DKI, diffusional kurtosis imaging; DTI, Diffusion tensor imaging; SPM, Statistical Parametric Mapping; VBM, Value based Management.

### Development and use of FMRI in MMD

The rs-fMRI technique is being used to study changes in functional connectivity patterns in patients with MMD by assessing the ALFF value of BOLD activity in the resting state of the task (34). Yu Lei et al. found for the first time that there is a corresponding change in the value of ALFF in adult patients with vascular cognitive impairment of MMD (35).

Their study found that there were wide differences in ALFF in frontal lobe, parietal lobe and temporal lobe between the designed vascular cognitive impairment group, the non-vascular cognitive impairment group and the control group, and there were significant differences in ALFF in the anterior cingulate cortex and the right auxiliary motor area of the frontal lobe. In the process of progressive cognitive decline in MMD patients, ALFF in parietal gyrus, right superior frontal gyrus, right superior temporal gyrus, left caudate nucleus and other regions showed significant changes. Moreover, they proposed that patients with MMD may have special spatial patterns of ALFF, and the changes of these patterns occurred after the emergence of cognitive impairment (35).

The team also found abnormal regional homogeneity (Reho) in executive control networks (ECN), default mode networks (DMN), and salience networks (SN) in adult patients with MMD. Compared with normal controls, patients with MMD exhibited significantly decreased ReHo in the dorsolateral prefrontal cortex (DLPFC) and inferior parietal gyrus (IPG) of left ECN; the IPG, superior frontal gyrus, and DLPFC of the right ECN; the right precuneus, left medial superior frontal gyrus, and right medial orbitofrontal gyrus of the DMN; as well as the left middle frontal gyrus and right supplemental motor area of SN. And a trend of ReHo decrease with disease severity was observed in these three networks, but only bilateral ECN reached statistical significance (36). And they highlighted that bilateral ECN exhibited a significant correlation of averaged ReHo values with executive performance. Similar finding has also been confirmed in other studies. He SH et al. also found decreased activation in the posterior cingulate gyrus, the left superior parietal gyrus, and the left superior occipital gyrus in the right ECN (37). And they indicated that decreased computational ability in patients with MMD was associated with significant abnormalities in the CBF of the left inferior frontal gyrus.

Sakamoto et al. found that DMN connectivity have changed in patients with MMD, their results showed highly disrupted patterns of ventral DMN connectivity, with a mixture of higher and lower functional connectivity in patients with low neuropsychologic scores compared with healthy controls (6). He et al. (37) also found that there were significantly fewer functional connections in the brain in the asymptomatic MMD group than in the control group. Furthermore, a study designed by Lei (38) introduced a dynamic measurement of connectivity number entropy (CNE) to further explore the relationship between brain networks and vascular cognitive impairment (VCI) in patients with MMD, they found that only the ECN and DMN exhibited statistical CNE differences among the three groups (VCI, VCI with intact cognition, normal controls), implying their cognitive-related significance.

Some studies have involved both white matter damage and functional connectivity. Kazumata used graph theoretical analysis to study the relationship between covert white matter injury and abnormal brain network characteristics. The results showed that global network parameters were reduced in patients with MMD, including cluster coefficient, characteristic path length, and small-world property. Reduced pairwise connectivity was found in prefrontal neural circuits within the middle/inferior frontal gyrus; supplementary motor area; and insular, inferior temporal, and dorsal cingulate cortices (39). Similarly, Hu (40) researched that whether the impaired functional connectivity and cognitive performances were attributed to the destruction of white matter fibers, the results also showed that there was lower functional connectivity in MMD patients as compared to HCs between the left supplementary motor area and inferior frontal gyrus, which is correlated with incomplete integrity of white matter fibers, and may contribute to impaired cognitive performance. These studies combined DTI and rs-fMRI techniques and could be useful in the evaluation of disease progression and prognosis of MMD.

It is worth noting that a study by Kazumata et al. (6, 31) showed that such change in functional connectivity is related to certain clinical features, depending on the corresponding damaged anatomical functional areas, and can be improved after revascularization surgery. More article details could be found in Table 2.

**Table 2:** Studies in recent years about rs-fMRI analysis of MMD.

**Main topic**	**Time**	**Author**	**Key point**	**Observe indicators**	**Analysis method**	**Other design methods**	**Patient selection**
Local brain activity	2014	Yu Lei	MMD patients exhibit a specific pattern of ALFF and that this pattern changes following cognitive impairment.	ALFF	SPM8	Cognition test	11 with VCI and 12 without VCI (NonVCI)
	2016	Yu Lei	Aberrant ReHo of ECN, DMN, SN exists in MMD patients with executive dysfunction.	ReHo	SPM8	Cognition test	26 without infarction
	2021	Shihao He	There are differences in the posterior cingulate gyrus, the left superior parietal gyrus, and the left superior occipital gyrus of the ECN.	ICA	SPM8	CBF, Cognition test	26 without infarction
Brain functional connectivity	2018	Yusuke Sakamoto	There are marked changes in FC of the ventral DMN of MMD patients with low cognitive ability scores, and it can be improved after surgery.	FC	SPM8, FSL	Cognition test	7 patients
	2022	Junwen Hu	There are abnormal brain FC between the left supplementary motor area and inferior frontal gyrus in MMD.	FC	CONN(ROI-to-ROI)	DTI, Cognition test	22 patients
Brain functional networks	2015	Ken Kazumata	Graph theoretical analysis was used to found that global network parameters were reduced in patients with MMD	topologic properties	FSL-TBSS, BCT	DTI, Cognition test	23 asymmetric
	2020	Yu Lei	Introduced a dynamic measurement of connectivity number entropy (CNE) to characterize both spatial and temporal dimensions of network interactions	CNE, topologic properties	SPM12, BCT	Cognition test	52 patients

>ECN, executive control networks; DMN, default mode networks; SN, salience networks; ALFF, low frequency fluctuation amplitude; ReHo, regional homogeneity; VCI, vascular cognitive impairment; CBF, cerebral blood flow; FC, Functional Connectivity; TBSS, Tract-Based Spatial Statistics; DTI, Diffusion tensor imaging; SPM, Statistical Parametric Mapping; ICA, independent component analysis; BCT, Brain Connectivity Toolbox.

## Difficulty and challenge

### Difficulties and challenges of DTI in MMD

The limitation of DTI technology itself is actually the biggest difficulty for experimental design and deep discussion of results. First, the degree of myelination correlates with FA, but does not determine tissue anisotropy, as has been demonstrated in non-myelinated fibers. Since axon numbers and myelin are strongly correlated, they cannot be distinguished when discuss FA changes. Therefore, FA should not be equated with an indicator of myelination or myelination injury. Thirdly, the FA value is generally higher in the central area where white matter is concentrated, and lower in the peripheral area where white matter is relatively sparse. However, contradictory FA values will decrease in the area where white matter is crossed (41). This is due to the limitations of DTI model for fiber crossover. Therefore, some scholars used diffusional kurtosis imaging (DKI) technology to explore (42). While achieving similar results with DTI, they found that DKI can also show the microstructural changes of deep white matter prone to ischemia and solve the problem of fiber crossover. In addition, different rates of fiber development and degeneration also affect the measurement results of DTI (43). For example, the upper longitudinal bundle matures relatively late and FA values show a gradient decline in late adulthood (44, 45). These interferes in the design and discussion of DTI studies and are more restrictive to age matching and selection of patients.

### Difficulties and challenges of FMRI in MMD

It must be noted that although resting state fMRI is widely used in MMD, task-state functional magnetic resonance imaging (tasking-state fMRI, ts-fMRI) is rarely used in MMD, even though ts-fMRI is widely used in stroke-related studies (46–48). The reasons are as follows: ① Because BOLD signal indirectly reflects the activities of neurons through the changes of blood components, BOLD fMRI largely ignores the effects of abnormal vascular reactivity (CVR) and abnormal neurovascular coupling (49). ② In patients with MMD, the intima of the main artery is eccentrically thickened and the smooth muscle layer of the media becomes thinner due to the formation of a large number of fibers and smooth muscle cells; most of the lumens of the MMD vessels are enlarged and the walls of the vessels become thinner, and the internal elastic layer becomes thinner and even broken in patients with severe dilatation (50). ③ There are compensatory neovascularization in the medial Dura of patients with MMD, the intima of these vessels are very thin and markedly different from normal blood vessels (51), the elasticity and low resistance of new blood vessels make it easier for blood to flow into them, this can lead to “blood theft phenomenon” (52, 53). These changes lead to a decrease in CVR, so abnormal neurovascular coupling phenomenon leads to complexity of BOLD signal in MMD patients during task-state testing.

However, a recent study of Mazerolle discussed the effect of abnormal CVR on BOLD signal in MMD patients and proposed new insights. Their test results show that CVR damaged areas can still show increased BOLD signals to meet the needs of related tasks. Therefore, they believe that the value of regional CVR reflects not only the ability of local blood vessels to respond to neural activity, but also the net response of local blood vessels to brain activity as a whole (54). But the study included only two MMD patients, and further studies are needed to add to the evidence.

In future work, dealing with the vascular-neural coupling problem of rsfMRI is still a topic that needs to be discussed and improved, Interdisciplinary approaches in the field of network science can help solve the further problems of the dynamics, stability and interaction of these brain networks. Because of the high demand for FMRI data acquisition and analysis, transdisciplinarity and large scale data sharing activities are critical (55). Overcoming the difficulty of measuring perfusion changes with ASL in underperfused brain regions will also provide value for FMRI in MMD.

## Author contributions

XH, ZL, and SH wrote and edited the manuscript. YW, YZ, and RW were also involved in drafting the manuscript and revising it critically for important intellectual content. All authors contributed to the article and approved the submitted version.

## Funding

This study was supported partially by the National Natural Science Foundation of China (82171887).

## Conflict of interest

The authors declare that the research was conducted in the absence of any commercial or financial relationships that could be construed as a potential conflict of interest.

## Publisher's note

All claims expressed in this article are solely those of the authors and do not necessarily represent those of their affiliated organizations, or those of the publisher, the editors and the reviewers. Any product that may be evaluated in this article, or claim that may be made by its manufacturer, is not guaranteed or endorsed by the publisher.

## References

[1] Kuroda S Houkin K. Moyamoya disease: current concepts and future perspectives. Lancet Neurol. (2008) 7:1056–66. 10.1016/S1474-4422(08)70240-0 18940695

[2] Suzuki J Takaku A. Cerebrovascular “moyamoya” disease. Disease showing abnormal net-like vessels in base of brain. Arch Neurol. (1969) 20:288–99. 10.1001/archneur.1969.00480090076012 5775283

[3] Wang L Lin F Wu J Jiao Y Cao Y Zhao Y. Plasticity of motor function and surgical outcomes in patients with cerebral arteriovenous malformation involving primary motor area: insight from fMRI and DTI. Chin Neurosurg J. (2016) 2:12. 10.1186/s41016-016-0030-y

[4] Tong X Wu J Lin F Cao Y Zhao Y Jin Z. Involvement of the visual pathway is not a risk factor of visual field deficits in patients with occipital arteriovenous malformations: an fMRI study. Chin Neurosurg J. (2015) 1:10. 10.1186/s41016-015-0010-7

[5] Liu ZQ He SH Xu ZS Duan R Yuan L Xiao C. Association between white matter impairment and cognitive dysfunction in patients with ischemic Moyamoya disease. BMC Neurol. (2020) 20:302. 10.1186/s12883-020-01876-0 32799829 PMC7429789

[6] Sakamoto Y Okamoto S Maesawa S Bagarinao E Araki Y Izumi T. Default mode network changes in moyamoya disease before and after bypass surgery: preliminary report. World Neurosurg. (2018) 112:E652–61. 10.1016/j.wneu.2018.01.117 29374613

[7] Pinheiro GR Soares GS Costa AL Lotufo RA Rittner L. Divergence map from diffusion tensor imaging: concepts and application to corpus callosum. Annu Int Conf IEEE Eng Med Biol Soc. (2016) 2016:1120–3. 10.1109/EMBC.2016.7590900 28268522

[8] Basser PJ Pierpaoli C. Microstructural and physiological features of tissues elucidated by quantitative-diffusion-tensor MRI. J Magnet Reson Ser B. (1996) 111:209–19. 10.1006/jmrb.1996.0086 8661285

[9] Alexander AL Lee JE Lazar M Field AS. Diffusion tensor imaging of the brain. Neurotherapeutics. (2007) 4:316–29. 10.1016/j.nurt.2007.05.011 17599699 PMC2041910

[10] Feldman HM Yeatman JD Lee ES Barde LHF Gaman-Bean S. Diffusion tensor imaging: a review for pediatric researchers and clinicians. J Dev Behav Pediatr. (2010) 31:346–56. 10.1097/DBP.0b013e3181dcaa8b 20453582 PMC4245082

[11] Beaulieu C. The basis of anisotropic water diffusion in the nervous system – a technical review. NMR Biomed. (2002) 15:435–55. 10.1002/nbm.782 12489094

[12] Huppi PS Murphy B Maier SE Zientara GP Inder TE Barnes PD. Microstructural brain development after perinatal cerebral white matter injury assessed by diffusion tensor magnetic resonance imaging. Pediatrics. (2001) 107:455–60. 10.1542/peds.107.3.455 11230582

[13] Vangelderen P Devleeschouwer MHM Despres D Pekar J Vanzijl PCM Moonen CTW. Water diffusion and acute stroke. Magn Reson Med. (1994) 31:154–63. 10.1002/mrm.1910310209 8133751

[14] Ugurbil K. Development of functional imaging in the human brain (fMRI); the University of Minnesota experience. Neuroimage. (2012) 62:613–9. 10.1016/j.neuroimage.2012.01.135 22342875 PMC3530260

[15] Ogawa S Menon RS Kim SG Ugurbil K. On the characteristics of functional magnetic resonance imaging of the brain. Ann Rev Biophys Biomol Struct. (1998) 27:447–74. 10.1146/annurev.biophys.27.1.447 9646874

[16] Logothetis NK. The neural basis of the blood-oxygen-level-dependent functional magnetic resonance imaging signal. Philos Trans R Soc Lond B Biol Sci. (2002) 357:1003–37. 10.1098/rstb.2002.1114 12217171 PMC1693017

[17] Ogawa S Lee TM Kay AR Tank DW. Brain magnetic resonance imaging with contrast dependent on blood oxygenation. Proc Natl Acad Sci U S A. (1990) 87:9868–72. 10.1073/pnas.87.24.9868 2124706 PMC55275

[18] Zhu DJ Zhang T Jiang X Hu XT Chen HB Yang N. Fusing DTI and fMRI data: a survey of methods and applications. Neuroimage. (2014) 102:184–91. 10.1016/j.neuroimage.2013.09.071 24103849 PMC4012015

[19] Zou QH Zhu CZ Yang YH Zuo XN Long XY Cao QJ. An improved approach to detection of amplitude of low-frequency fluctuation (ALFF) for resting-state fMRI: fractional ALFF. J Neurosci Methods. (2008) 172:137–41. 10.1016/j.jneumeth.2008.04.012 18501969 PMC3902859

[20] Logothetis NK Pauls J Augath M Trinath T Oeltermann A. Neurophysiological investigation of the basis of the fMRI signal. Nature. (2001) 412:150–7. 10.1038/35084005 11449264

[21] Mohamed MA Yousem DM Tekes A Browner N Calhoun VD. Correlation between the amplitude of cortical activation and reaction time: a functional MRI study. Am J Roentgenol. (2004) 183:759–65. 10.2214/ajr.183.3.1830759 15333368

[22] Zang YF Jiang TZ Lu YL He Y Tian LX. Regional homogeneity approach to fMRI data analysis. Neuroimage. (2004) 22:394–400. 10.1016/j.neuroimage.2003.12.030 15110032

[23] Chen J Sun D Shi Y Jin W Wang Y Xi Q. Dynamic alterations in spontaneous neural activity in multiple brain networks in subacute stroke patients: a resting-state fMRI study. Front Neurosci. (2018) 12:994. 10.3389/fnins.2018.00994 30666181 PMC6330292

[24] Kang D-Z Chen F-X Chen F-Y Liu Y Wu G Yu L-H. Altered regional homogeneity of prefrontal cortex in Parkinson's disease with mild cognitive impairment. Chin Neurosurg J. (2016) 2:10. 10.1186/s41016-016-0028-5

[25] Horn A Ostwald D Reisert M Blankenburg F. The structural-functional connectome and the default mode network of the human brain. Neuroimage. (2014) 102:142–51. 10.1016/j.neuroimage.2013.09.069 24099851

[26] Mori N Miki Y Fushimi Y Kikuta K Urayama S Okada T. Cerebral infarction associated with moyamoya disease: histogram-based quantitative analysis of diffusion tensor imaging – a preliminary study. Magn Reson Imaging. (2008) 26:835–40. 10.1016/j.mri.2008.01.036 18467061

[27] Xu L Yang Y Guo Ea Tao X Lu T Tian R. Diagnostic evaluation of patients with disorders of consciousness with diffusion tensor imaging. Chin Neurosurg J. (2017) 3:17. 10.1186/s41016-017-0079-2

[28] Jeong H Kim J Choi HS Kim ES Kim DS Shim KW. Changes in integrity of normal-appearing white matter in patients with moyamoya disease: a diffusion tensor imaging study. Am J Neuroradiol. (2011) 32:1893–8. 10.3174/ajnr.A2683 21920867 PMC7966016

[29] Kazumata K Tha KK Narita H Kusumi I Shichinohe H Ito M. Chronic ischemia alters brain microstructural integrity and cognitive performance in adult moyamoya disease. Stroke. (2015) 46:354–60. 10.1161/STROKEAHA.114.007407 25538200

[30] Kazumata K Tha KK Uchino H Shiga T Shichinohe H Ito M. Topographic changes in cerebral blood flow and reduced white matter integrity in the first 2 weeks following revascularization surgery in adult moyamoya disease. J Neurosurg. (2017) 127:260–9. 10.3171/2016.6.JNS16653 27588593

[31] Kazumata K Tha KK Tokairin K Ito M Uchino H Kawabori M. Brain structure, connectivity, and cognitive changes following revascularization surgery in adult moyamoya disease. Neurosurgery. (2019) 85:E943–52. 10.1093/neuros/nyz176 31157394

[32] Fields RD. White matter matters. Sci Am. (2008) 298:54–61. 10.1038/scientificamerican0308-54 18357821

[33] Assaf Y Blumenfeld-Katzir T Yovel Y Basser PJ. AxCaliber: a method for measuring axon diameter distribution from diffusion MRI. Magn Reson Med. (2008) 59:1347–54. 10.1002/mrm.21577 18506799 PMC4667732

[34] Lehman VT Cogswell PM Rinaldo L Brinjikji W Huston J Klaas JP. Contemporary and emerging magnetic resonance imaging methods for evaluation of moyamoya disease. Neurosurg Focus. (2019) 47:E6. 10.3171/2019.9.FOCUS19616 31786551

[35] Lei Y Li Y Ni W Jiang H Yang Z Guo Q. Spontaneous brain activity in adult patients with moyamoya disease: a resting-state fMRI study. Brain Res. (2014) 1546:27–33. 10.1016/j.brainres.2013.12.022 24380677

[36] Lei Y Su JB Jiang HQ Guo QH Ni W Yang H. Aberrant regional homogeneity of resting-state executive control, default mode, and salience networks in adult patients with moyamoya disease. Brain Imaging Behav. (2017) 11:176–84. 10.1007/s11682-016-9518-5 26843005

[37] He SH Liu ZQ Wei YC Duan R Xu ZS Zhang C. Impairments in brain perfusion, executive control network, topological characteristics, and neurocognition in adult patients with asymptomatic Moyamoya disease. BMC Neurosci. (2021) 22:35. 10.1186/s12868-021-00638-z 33980154 PMC8117595

[38] Lei Y Song BS Chen L Su JB Zhang X Ni W. Reconfigured functional network dynamics in adult moyamoya disease: a resting-state fMRI study. Brain Imaging Behav. (2020) 14:715–27. 10.1007/s11682-018-0009-8 30511114

[39] Kazumata K Tha KK Narita H Shichinohe H Ito M Uchino H. Investigating brain network characteristics interrupted by covert white matter injury in patients with moyamoya disease: insights from graph theoretical analysis. World Neurosurg. (2016) 89:654–65.e2. 10.1016/j.wneu.2015.11.100 26724619

[40] Hu JW Li Y Li ZQ Chen JY Cao Y Xu D. Abnormal brain functional and structural connectivity between the left supplementary motor area and inferior frontal gyrus in moyamoya disease. BMC Neurol. (2022) 22:179. 10.1186/s12883-022-02705-2 35578209 PMC9108139

[41] Zhai GH Lin WL Wilber KP Gerig G Gilmore JH. Comparisons of regional white matter diffusion in healthy neonates and adults performed with a 3.0-T head-only MR imaging unit. Radiology. (2003) 229:673–81. 10.1148/radiol.2293021462 14657305

[42] Kazumata K Tha KK Narita H Ito YM Shichinohe H Ito M. Characteristics of diffusional kurtosis in chronic ischemia of adult moyamoya disease: comparing diffusional kurtosis and diffusion tensor imaging. Am J Neuroradiol. (2016) 37:1432–9. 10.3174/ajnr.A4728 27012294 PMC7960286

[43] Lebel C Walker L Leemans A Phillips L Beaulieu C. Microstructural maturation of the human brain from childhood to adulthood. Neuroimage. (2008) 40:1044–55. 10.1016/j.neuroimage.2007.12.053 18295509

[44] Sullivan EV Rohlfing T Pfefferbaum A. Longitudinal study of callosal microstructure in the normal adult aging brain using quantitative DTI fiber tracking. Dev Neuropsychol. (2010) 35:233–56. 10.1080/87565641003689556 20446131 PMC2867078

[45] Huang H Zhang JY Wakana S Zhang WH Ren TB Richards LJ. White and gray matter development in human fetal, newborn and pediatric brains. Neuroimage. (2006) 33:27–38. 10.1016/j.neuroimage.2006.06.009 16905335

[46] Bergfeldt U Jonsson T Bergfeldt L Julin P. Cortical activation changes and improved motor function in stroke patients after focal spasticity therapy-an interventional study applying repeated fMRI. BMC Neurol. (2015) 15:52. 10.1186/s12883-015-0306-4 25884323 PMC4450484

[47] Richards LG Stewart KC Woodbury ML Senesac C Cauraugh JH. Movement-dependent stroke recovery: a systematic review and meta-analysis of TMS and fMR1 evidence. Neuropsychologia. (2008) 46:3–11. 10.1016/j.neuropsychologia.2007.08.013 17904594 PMC2248459

[48] Stinear C. Prediction of recovery of motor function after stroke. Lancet Neurol. (2010) 9:1228–32. 10.1016/S1474-4422(10)70247-7 21035399

[49] Pike GB. Quantitative functional MRI: concepts, issues and future challenges. Neuroimage. (2012) 62:1234–40. 10.1016/j.neuroimage.2011.10.046 22056462

[50] Rao ML Zhang H Liu Q Zhang SQ Hu LS Deng F. Clinical and experimental pathology of Moyamoya disease. Chin Med J. (2003). 116:1845–9. 14687471

[51] Mukawa M Nariai T Inaji M Tamada N Maehara T Matsushima Y. First autopsy analysis of a neovascularized arterial network induced by indirect bypass surgery for moyamoya disease: case report. J Neurosurg. (2016) 124:1211–4. 10.3171/2015.4.JNS15155 26406800

[52] Sobczyk O Battisti-Charbonney A Fierstra J Mandell DM Poublanc J Crawley AP. A conceptual model for CO2-induced redistribution of cerebral blood flow with experimental confirmation using BOLD MRI. Neuroimage. (2014) 92:56–68. 10.1016/j.neuroimage.2014.01.051 24508647

[53] Mikulis DJ. Chronic neurovascular uncoupling syndrome. Stroke. (2013) 44:S55–7. 10.1161/STROKEAHA.113.001081 23709731

[54] Mazerolle EL Ma Y Sinclair D Pike GB. Impact of abnormal cerebrovascular reactivity on BOLD fMRI: a preliminary investigation of moyamoya disease. Clin Physiol Funct Imaging. (2018) 38:87–92. 10.1111/cpf.12387 27572110 PMC5763346

[55] Yang J Gohel S Vachha B. Current methods and new directions in resting state fMRI. Clin Imaging. (2020) 65:47–53. 10.1016/j.clinimag.2020.04.004 32353718 PMC7365764

